# Exogenous Application of Phytohormones Promotes Growth and Regulates Expression of Wood Formation-Related Genes in *Populus simonii* × *P. nigra*

**DOI:** 10.3390/ijms20030792

**Published:** 2019-02-12

**Authors:** Hongmei Yuan, Lijuan Zhao, Wendong Guo, Ying Yu, Lei Tao, Liguo Zhang, Xixia Song, Wengong Huang, Lili Cheng, Jing Chen, Fengzhi Guan, Guangwen Wu, Huiyu Li

**Affiliations:** 1 Heilongjiang Academy of Agricultural Sciences Postdoctoral Programme, 368 Xuefu Road, Harbin 150086, China; yuanhm1979@163.com (H.Y.); zhangliguohemp@163.com (L.Z.); 2 Industrial Crops Institute, Heilongjiang Academy of Agricultural Sciences, 368 Xuefu Road, Harbin 150086, China; yuying_1981_0451@163.com (Y.Y.); jjzwyjs@163.com (X.S.); huangwengong1736@163.com (W.H.); chenglili_i@163.com (L.C.); ccyj15@163.com (J.C.); guanfengzhiflax@163.com (F.G.); 3 State Key Laboratory of Tree Genetics and Breeding, Northeast Forestry University, 26 Hexing Road, Harbin 150040, China; 4 Crop Breeding Institute, Heilongjiang Academy of Agricultural Sciences, 368 Xuefu Road, Harbin 150086, China; zlj-110@163.com; 5 Institute of Natural Resources and Ecology, Heilongjiang Academy of Sciences, 103 Haping Road, Harbin 150040, China; guowendong988@163.com; 6 College of Life Science, Northeast Forestry University, 26 Hexing Road, Harbin 150040, China; taolei2003-01@163.com

**Keywords:** *Populus simonii* × *P. nigra*, phytohormone, wood formation related gene, expression profiles, qPCR

## Abstract

Although phytohormones are known to be important signal molecules involved in wood formation, their roles are still largely unclear. Here, *Populus simonii* × *P. nigra* seedlings were treated with different concentrations of exogenous phytohormones, indole-3-acetic acid (IAA), gibberellin (GA_3_), and brassinosteroid (BR), and the effects of phytohormones on growth were investigated. Next, 27 genes with known roles in wood formation were selected for qPCR analysis to determine tissue-specificity and timing of responses to phytohormone treatments. Compared to the control, most IAA, GA_3_, and BR concentrations significantly increased seedling height. Meanwhile, IAA induced significant seedling stem diameter and cellulose content increases that peaked at 3 and 30 mg·L^−1^, respectively. Significant increase in cellulose content was also observed in seedlings treated with 100 mg·L^−1^ GA_3_. Neither stem diameter nor cellulose content of seedlings were affected by BR treatment significantly, although slight effects were observed. Anatomical measurements demonstrated improved xylem, but not phloem, development in IAA- and BR-treated seedlings. Most gene expression patterns induced by IAA, GA_3_, and BR differed among tissues. Many IAA response genes were also regulated by GA_3_, while BR-induced transcription was weaker and slower in *Populus* than for IAA and GA_3_. These results reveal the roles played by phytohormones in plant growth and lay the foundation for exploring molecular regulatory mechanisms of wood formation in *Populus*.

## 1. Introduction

Wood, or secondary xylem, is a water-conductive and supportive vascular tissue that is predominantly found in trees. Wood has uses in construction, pulp, and paper production and in the future will likely play a major role in production of biofuels—a renewable, cost-effective alternative to fossil fuels [1]. The formation of both primary and secondary xylem involves a cascade of interesting processes, including arrangement of primary vascular tissue into bundles, cell proliferation within primary bundles or in secondary vascular cambium, initiation of xylem differentiation, regulation of cell expansion, deposition of a secondary cell wall, and programmed cell death [2]. New insights are coming to light regarding xylem differentiation [3,4], a process signaled and directed by hormonal signaling cascades. Although signaling pathways for some of these compounds are quite well characterized (such as for auxin, cytokinin, gibberellin and brassinosteroid), the detailed molecular mechanisms underlying their control of vascular development are not fully understood [3,5,6].

Wood formation occurs during the secondary phase of plant development. Wood biomass is mainly made up of secondary walls which are composed of three major biopolymers: cellulose, hemicelluloses and lignin. Formation of secondary walls is a complex process that requires the coordinated expression of secondary wall-specific biosynthetic genes to direct biosynthesis and targeted secretion of secondary wall components followed by patterned deposition and assembly of components to form final secondary wall structures [7]. Most genes involved in biosynthesis of secondary wall components, including cellulose, xylan, glucomannan and lignin, have been identified and their coordinated activation is mediated by a transcriptional network involving secondary wall NAM–ATAF1,2–CUC2 (NAC) and myeloblastosis (MYB) master switches and downstream transcription factors [7]. Moreover, proteins encoded by these genes have also been shown to be involved in hormone metabolism, transport and signaling, among other functions. However, our understanding of how vascular tissue develops remains far from complete [1]. Elucidation of mechanisms underlying the coordinated activation of secondary wall biosynthetic genes will undoubtedly augment our understanding with regard to the molecular regulation of wood formation [8].

Phytohormones regulate essential physiological and developmental processes during a plant’s life cycle [9,10]. Earlier studies had shown that indole-3-acetic acid (IAA) plays an important role in cambial activity and wood formation of woody plants [11,12]. Since then, numerous experiments using exogenous auxin treatments of both hardwoods and conifers have demonstrated the potential of IAA to affect most aspects of cambial growth in a dose-dependent manner, including xylem and phloem production and size, as well as secondary wall thickness of xylem elements [13,14,15,16]. Gibberellins (GAs) are also important regulators of stem growth and wood formation and considerable evidence demonstrates that exogenous application of gibberellic acid (GA_3_) promotes cambial cell division, expansion of cambium derivatives and tension wood formation [5,17,18,19]. More recently, brassinosteroids (BRs) have also been identified as essential regulators of vascular development [3,6,20]. BRs are produced in procambial cells and trigger xylem precursor cells to induce xylem differentiation. Notably, exogenously supplied BRs elicit diverse biological activities, including stem elongation and vascular differentiation [21,22]. IAA, GA_3_, and BR are all important signal molecules that play roles in induction of wood formation. However, in spite of the aforementioned body of knowledge, our understanding of how wood formation regulated by the phytohormones is still limited.

Poplar is a model species for studies of angiosperm trees, particularly because the entire genome of *Populus trichocarpa* has been sequenced. In the present study, we investigated the effects of IAA, GA_3_, and BR on growth of Xiaohei poplars (*Populus simonii* × *P. nigra*), including effects on plant height, stem diameter, cellulose content and xylem development. In addition, 27 genes related to wood formation in poplars were selected and studied using tissue-specific and time-series analyses of transcriptional responsiveness to the three phytohormones [1]. This study aimed to determine (1) which hormone plays the most prominent role in regulation of growth and gene expression; (2) characteristics of tissue-specific regulation of genes in response to IAA, GA_3_ and BR; (3) whether the three phytohormones function coordinately or antagonistically during growth regulation of poplar. The results provide insights into the roles played by phytohormones in plant growth and lay the foundation for exploring the molecular regulatory mechanisms of wood formation in poplars.

## 2. Results

### 2.1. Exogenous Phytohormones Promote Plant Growth and Cellulose Synthesis

**Figure 1:**
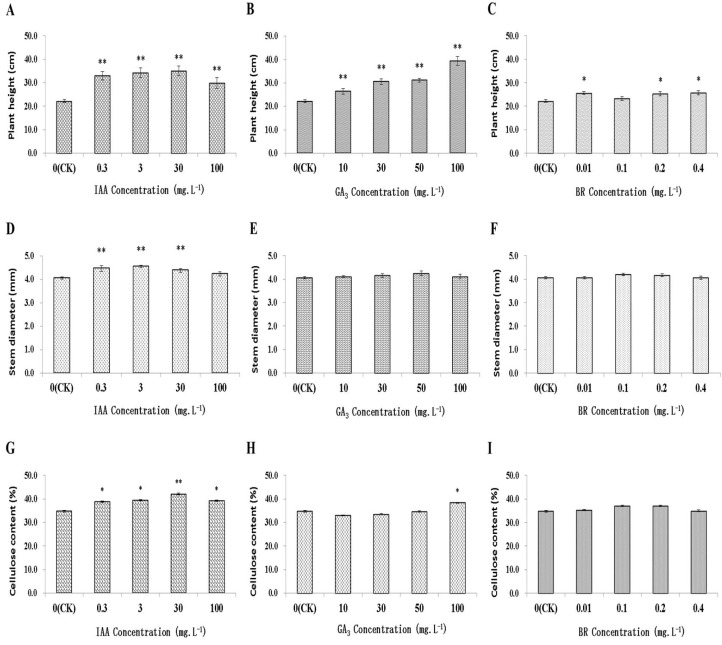
Effects of exogenously applied indole-3-acetic acid (IAA), gibberellin (GA_3_) and brassinosteroid (BR) on plant growth and cellulose synthesis: plant height (**A**–**C**), stem diameter (**D**–**F**) and cellulose content (**G**–**I**). * and ** indicate significant differences at *p* < 0.05 and *p* < 0.01.

To determine whether exogenous phytohormones affect normal growth and development of *Populus*, seedlings were treated with various concentrations of exogenously provided IAA, GA_3_ and BR. As shown in Figure 1, after four months of treatment, seedlings exhibited fast-growing phenotypes with greater plant height observed for almost all concentrations of IAA, GA_3_, and BR than for the untreated control. The average plant height and stem diameter of control trees were 22.23 cm and 4.08 mm, respectively. Seedlings treated with 100 mg·L^−1^ GA_3_ attained the greatest height (39.38 cm), a value 77.15% higher than the average control height. Meanwhile, significant increases in average stem diameter were observed in IAA treated plants, with the maximum response at 3 mg·L^−1^, resulting in an average diameter that was 10.78% greater than that of the control. While only insignificant and slightly increased stem diameters were observed for GA_3_ and BR treatments compared to control (*p* > 0.05). Cellulose content ranged from 33.08% to 42.14%. Significant increases in cellulose content were observed in IAA treated plants, with a peak response at 30 mg·L^−1^ exhibiting a cellulose content value of 21.09% greater than the control value (*p* < 0.01). Cellulose content values of seedlings were only slightly affected by BR treatment, with results not attaining significance (*p* > 0.05). Collectively, the results suggest that IAA affected seedling growth more profoundly than did treatments with GA_3_ or BR in *Populus*.

### 2.2. Phytohormones Promote Xylem Differentiation in Populus

**Figure 2:**
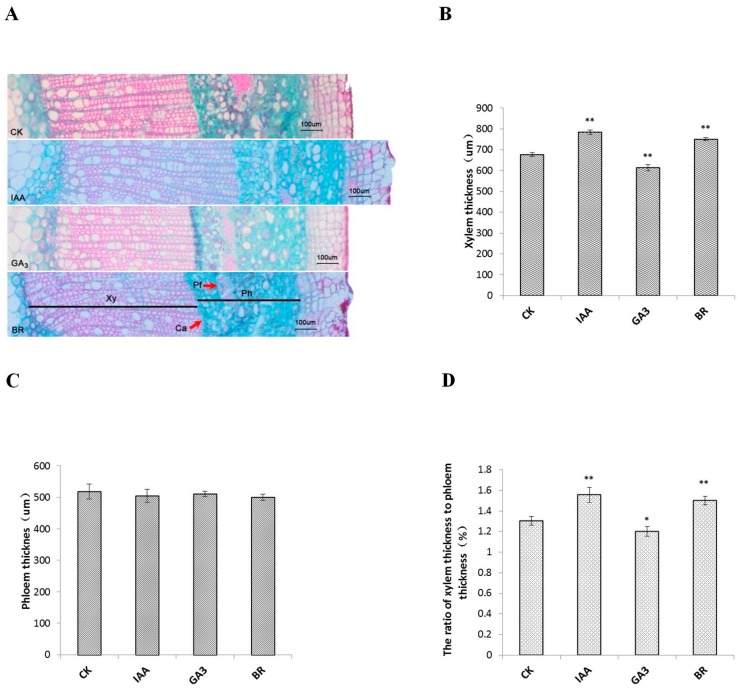
Effects of phytohormones on xylem and phloem differentiation in 5-month-old *Populus*. (**A**) Cross sections of stems in phytohormone-treated and untreated plants. (**B**) Measurement of xylem thickness. (**C**) Measurement of phloem thickness. (**D**) The ratio of xylem thickness to phloem thickness. * and ** indicate significant differences in comparison with control at *p* < 0.05 and *p* < 0.01, respectively. Cambial zone (Ca), Phloem (Ph), Phloem fiber (Pf), Xylem (Xy), Bar = 100 µm.

To further understand the potential function of phytohormones during secondary growth of woody plants, stems of control and experimental seedlings treated with IAA, GA_3_, and BR were measured to determine anatomical characteristics (Figure 2A). Xylem thickness improved significantly in IAA- and BR-treated seedlings, due to the enlargement of intercellular spaces (Figure 2B), while no significant differences were seen for phloem (Figure 2C). Meanwhile, xylem and phloem developed significantly slower in GA_3_-treated seedlings than in controls (*p* < 0.01). The ratio of xylem thickness to phloem thickness was also calculated, respectively (Figure 2D) and ratios of 1.56 and 1.50, respectively, for IAA- and BR-treated seedlings were observed that were significantly higher than the control ratio. However, a significant decrease in ratio was observed for GA_3_ treatment compared to control (*p* < 0.05).

### 2.3. Expression Profiles of Genes in Response to IAA, GA_3_, and BR Treatments

Phytohormone responses of 27 wood formation related genes in *Populus simonii* × *P. nigra* were analyzed using qRT-PCR (Table 1). Genes were chosen based on their involvement in cell wall biosynthesis, transcription regulation, phytohormone biosynthesis and signaling pathways. The level of significance was set to |log_2_^f^°^ld change^| >1 and *p* < 0.05. Log_2_^fold change^ values were shown in Tables S1–S3. The effects of the three hormones on the expression of the 27 studied genes were shown in Table S4.

**Figure 3:**
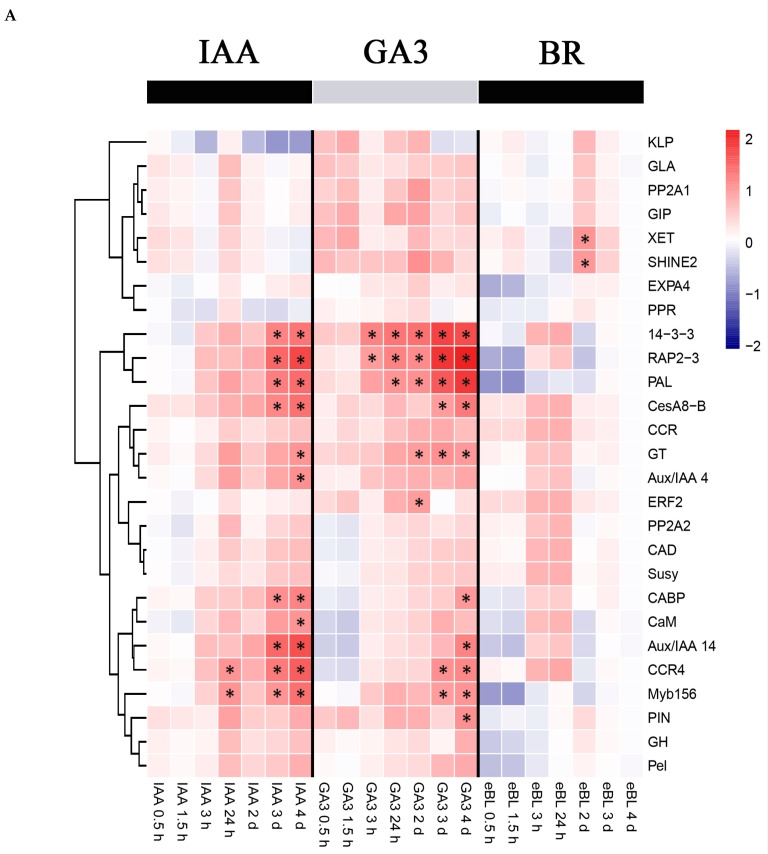

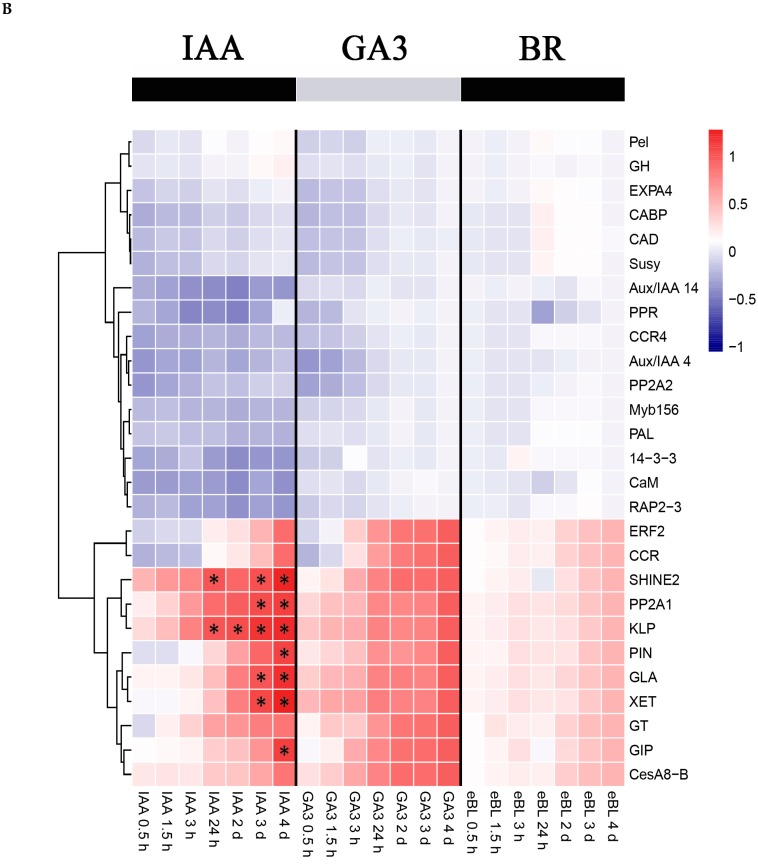

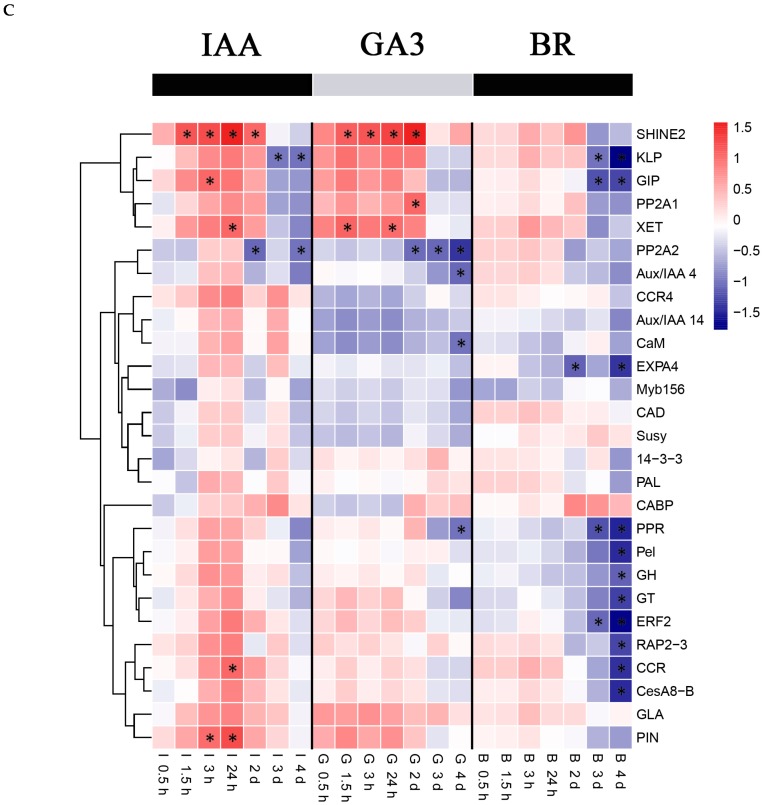
Hierarchical clustering of gene expression by qPCR under IAA, GA_3_, and BR treatments. Treatment times are indicated at the bottom of the figure. The samples were harvested at time points of 0.5 h, 1.5 h, 3 h, 24 h, 2 d, 3 d and 4 d. (**A**) Stem, (**B**) Root, (**C**) Leaf. * indicates significant differences in comparison with control (|log_2_^f^°^ld change^| > 1 and *p* < 0.05).

In stems, most genes showed similar expression patterns under IAA and GA_3_ treatments (Figure 3A). Nine of these genes were significantly up-regulated in response to either IAA or GA_3,_ including *14-3-3*, *RAP2-3*, *PAL*, *CesA8-B*, *GT*, *CABP*, *Aux/IAA 14*, *CCR4* and *Myb156*. *Aux/IAA 4* and *CaM* were significantly up-regulated only under IAA treatment, while *ERF2* and *PIN* were significantly up-regulated only under GA_3_ treatment. Under BR treatment, only *XET* and *SHINE2* were significantly up-regulated.

In roots, the expression level of the genes changed slightly after treated with the three hormones at different time points. Seven of these genes were significantly up-regulated in response to IAA treatments, including *SHINE2*, *PP2A1*, *KLP*, *PIN*, *GLA*, *ExpA4*, *GIP* (Figure 3B). These genes were also up-regulated under both GA_3_ and BR treatments, while the differences did not reach statistical significance. It’s interesting that most genes showed a similar trend under the three hormone treatments.

In leaves, the genes showed complex expression patterns under the three hormone treatments (Figure 3C). Most genes were up-regulated at the early stage, then were gradually down-regulated at the late stage. The transcription factor *SHINE2* was dramatically induced by IAA and GA_3_. Genes involved in signal transduction were also significantly induced or repressed. *GIP* and *PIN* were significantly up-regulated in IAA-treated samples. *PP2A1* was significantly induced, while *PP2A2, Aux*/*IAA 4* and *CaM* were significantly repressed in GA_3_-treated samples. Meanwhile, expression levels of most genes involved in cell wall biosynthesis, such as *Klp*, *ExpA4*, *Pel*, *GH*, *GT*, *CCR* and *CesA8-B* were significantly down-regulated under BR treatment.

### 2.4. Correlation Network Analysis of Genes in Response to IAA, GA_3_, and BR Treatments

**Figure 4:**
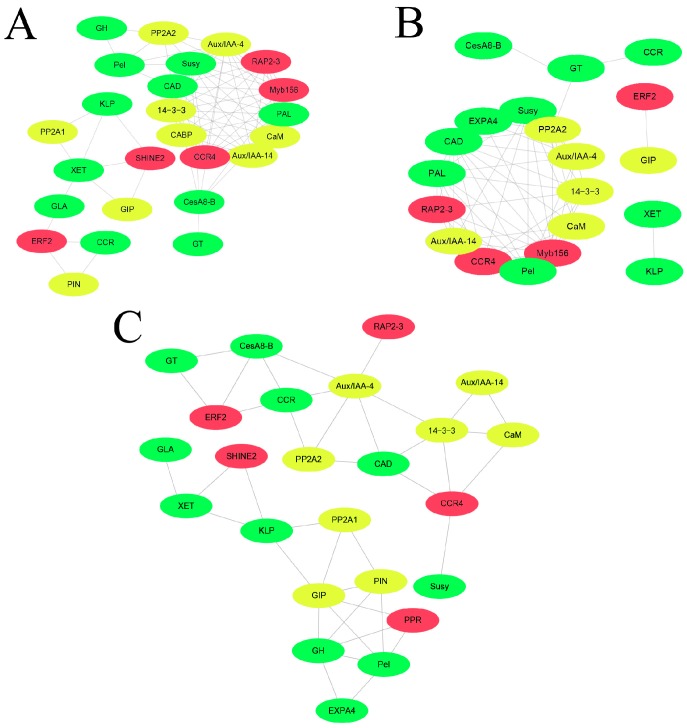
Correlation network of genes in response to phytohormones. (**A**) IAA, (**B**) GA_3_, (**C**) BR. Each node represents a cell wall biosynthesis-related gene (green), a transcription factor (red) or a signal transduction-related gene (yellow).

In this study, correlation network analysis was performed to assess potential gene interactions under IAA, GA_3_, and BR treatments. Table S5 lists each differentially expressed gene within the correlation network that exhibited a correlation coefficient > 0.85. Co-expression network analysis for IAA- and GA_3_-treated samples resulted in tightly co-expressed modular networks consisting of 25 nodes with 81 edges and 20 nodes with 61 edges, respectively (Figure 4A, 4B). Co-expression networks for BR-treated samples consisted of 24 nodes with 40 edges, with no tightly co-expressed modular network found (Figure 4C). Ultimately, all three modular networks shared ten genes, *Aux*/*IAA 4*, *Aux*/*IAA 14*, *CAD*, *CaM*, *CCR4*, *CesA8-B*, *PP2A2*, *RAP2-3*, *Susy* and *14-3-3*, that were strongly co-expressed. These commonly co-expressed genes may participate in important interactions and will be investigated further.

## 3. Discussion

The formation of secondary xylem (wood) and phloem is initiated in the vascular cambium [23]. Auxin is required for cambial growth and its concentration gradient across the cambial tissues has been suggested to provide positional information in wood development [15]. Perturbing auxin signaling by reducing auxin responsiveness reduced the cambial cell division activity, caused spatial deregulation of cell division of the cambial initials, and led to reductions in not only radial but also axial dimensions of fibers and vessels [24]. Our data were consistent with the previous studies that the xylem-to-phloem ratios were significantly increased in the IAA-treated seedlings compared to controls. However, there were no significant differences in phloem width between the IAA-treated seedlings and controls. Thus, the exogenous IAA appeared to affect xylem development much more than phloem development. The gibberellin (GA) plays a role in the processes of expansion or elongation of xylem elements [14,15,25,26]. Eriksson et al. (2000) found that increased GA biosynthesis in transgenic hybrid aspen trees promoted growth, biomass production and xylem fiber length [5]. Another study demonstrated that application of a GA biosynthesis inhibitor to wood-forming tissues of *Eucalyptus globulus* resulted in decreased GA levels and shorter xylem fibers, with no effect on radial width of fibers observed by Ridoutt et al. (1998) [27]. In the current work, our results demonstrated that GA promoted both radial growth and increased height. However, it should be noted that both xylem thickness and xylem to phloem ratio were lower than corresponding control values, while phloem thickness remained unaffected. This result is reminiscent of previous studies that the level of GA was very precisely controlled to keep the phloem to xylem ratio low. Too high levels of GA might change the balance between IAA and GA and thus the xylem to phloem ratio [28]. Taken together, these results suggest that the main effect of GA may be on xylem fiber elongation rather than on radial expansion. Brassinosteroid (BR) signaling has a well-established role in regulating primary vascular patterning in the *Arabidopsis* shoot [29]. A recent study indicated that brassinosteroid signaling may contribute to the regulation of secondary xylem production [30,31,32]. In the present study, significant increases in plant height and xylem thickness were observed in BR-treated plants. The growth promotion in BR-treated stems might be achieved by cell elongation and by accelerating cell division with preferential increase in fiber cell types [33]. However, in this study seedling cellulose content was at most slightly affected by BR treatments, as effects were not significant (*p* > 0.05). Our result conflicts with results of a previous study showing that BR up-regulated cellulose biosynthesis in *Arabidopsis* [34]. While the results of this work are consistent with results of a study of *Liriodendron* showing that BR application resulted in increased amounts of non-cellulosic cell wall carbohydrates, such as hemicellulose and pectin, rather than cellulose [33]. Taken together, in different plants BR effects on cell wall components may differ, but overall it appears that BR does affect cell wall integrity in plants [35,36].

Wood formation is a developmental process that involves highly coordinated expression of cell wall biosynthesis genes. Expression of *CesA8-B*, previously shown to be primarily responsible for synthesis of secondary cell wall cellulose [37], was shown here to significantly correlate positively with cellulose content. Genes encoding sucrose synthases (*Susy*) have also been implicated in cell wall biosynthesis, since overexpression of cotton *Susy* in poplar was shown to increase cellulose synthesis [38], although *Susy* expression was unaffected by IAA/GA_3_/BR treatment in the current study. Moreover, genes encoding cinnamoyl-CoA reductase (CCR) and cinnamyl alcohol dehydrogenase (CAD) may also participate in cell wall synthesis by encoding enzymes that catalyse two key reduction reactions involved in conversion of cinnamic acid derivatives into monolignol building blocks of cell wall lignin polymers [39]. Because our data showed that *CCR* expression in leaves was up-regulated by IAA and down-regulated by BR, while *CAD* expression was unaffected by IAA/GA3/BR treatments, *CCR* and *CAD* may be independently regulated within separate monolignol biosynthesis pathways. Meanwhile, genes encoding expansins (EXPs) and glycosyl hydrolases (GH) may also play roles in cell wall biosynthesis, with the former inducing cell wall extension during plant cell growth and the latter participating in physiologically important plant processes, such as activation of phytohormones, lignification and cell wall remodelling [40]. In this study, BR inhibition of *EXPA4* and *GH* transcription was observed, with expression of both genes unaffected by IAA or GA. KLP (kinesin-like protein) is essential for the oriented deposition of cellulose microfibrils and cell wall strength [41]. Our results indicated that *KLP* may be regulated by IAA, GA_3_ and BR, but it exhibits different expression patterns in leaves, stems and roots. Another important gene, *Pel* gene for the enzyme pectatelyase that catalyzes homogalacturon degradation, expressed at the onset of secondary cell wall formation when enzyme production is needed to increase solubility of wood matrix polysaccharides [42]. Our result showed that the gene was down-regulated by exogenous BR in leaves. Finally, expression of xyloglucan endotransglycosylases, (XET), which modify the xyloglucan-cellulose framework of plant cell walls, appears important for regulating cell wall expansion and strength [43]. In our study, IAA/GA_3_/BR could enhance *XET* expression, although phytohormone treatment effects differed among various tissues.

Wood formation is known to be regulated by a cascade of transcription factors (TFs). Among these, a set of MYB TFs that are functional orthologs of *Arabidopsis* TFs have been shown to be involved in the regulation of cell wall biosynthesis during wood formation in *Populus* [44,45,46]. Here we found a positive relationship between the expression of *Myb156* and *PAL*, suggesting that *Myb156* plays a role in lignin synthesis in *Populus*. Recently, AP2/EREBP domain transcription factors also have been found to participate in secondary wall formation in stem development of *Medicago truncatula*. Liu et al. (2017) identified a *Populus* AP2/ERF type transcription factor gene, *PsnSHN2* [47]. It activated or repressed promoter activities of transcription factors involved in secondary wall biosynthesis and acted as a highly hierarchical transcriptional activator. Meanwhile, our results revealed that AP2/EREBP domain transcription factors *RAP2-3* and *SHINE2* exhibited 3~4 fold increased expression levels in response to IAA or GA_3_ treatments. Thus, it has been suggested that AP2/EREBP domain transcription factors may be key transcription factors that coordinately regulate secondary wall biosynthesis. PPR proteins associate with transcription and translation machineries and are involved in various aspects of organellar mRNA processing, including splicing, cleavage and editing [48,49]. It was reported that *PPR* gene (Gh_A03G0489) was involved in the cotton fiber cell wall thickening process [50]. Here we found that the transcript level of *PPR* were decreased by GA_3_ and BR in leaves. The *PPR* gene had not been well characterized in plants. We speculated that *PPR* might be a promising candidate gene involved in hormone signal transduction and modulated cell wall biosynthesis.

During the last two decades, extensive lists of genes involved in hormone synthesis, catabolism and signal transduction have been compiled from data generated from mutant studies. However, details on how these genes are regulated remain unclear [51,52,53]. AUX/IAA proteins play central roles in auxin signal transduction [54,55], results consistent with high-level expression of *Aux/IAA 4* and *Aux/IAA* *14* observed in stems treated with IAA in this study. Because these two *Aux/IAA* members exhibited differing expression patterns in stem versus root in response to GA_3_, these results suggest that these genes may be differentially regulated in different tissues. Because other studies had implicated polar auxin transport in vessel differentiation and spatial patterning during secondary growth in *Populus* [56], we studied the roles of auxin transport proteins known as PIN-formed proteins (PINs) in this process due to their demonstrated effects on auxin distribution [57,58,59]. More specifically, Björklund et al. have shown that GA can activate IAA signaling in cambium by promoting expression of *PIN1* in cells in early xylem differentiation stages [9], while Li et al. (2005) demonstrated that BR application enhances polar auxin transport and endogenous auxin distribution by up-regulating *PIN* expression [60]. Our data agreed with both results in that *PIN* was down-regulated by BR in leaves, but up-regulated by GA_3_ in stems. Because phosphorylation dynamics of PIN proteins have been shown to be affected by protein phosphatase 2A (PP2A) and PINOID kinase, which act antagonistically to mediate their apical-basal polar delivery [61,62], we looked for similar effects in the present study. Indeed, our results demonstrated that similar patterns were observed regarding expression of *PIN* and *PP2A1* genes, indicating that *PIN* and *PP2A1* may be co-regulated by phytohormones at the transcriptional level. Therefore, it appears that the uneven distribution of auxin resulting from polar intercellular auxin transport in plant tissues may trigger a wide range of developmental processes, including vascular tissue differentiation and wood formation. 14-3-3 proteins participated in diverse signal-transduction pathways involved in phytohormone action by interacting with various regulatory proteins [63]. Calcium is a well-known component of the signal transduction pathway in activating and regulating numerous cellular processes. Our data suggested that the expression patterns of *14-3-3* share high similarity with those of *CaM* and *CABP* (Ca^2+^-binding proteins). Based on the findings mentioned above, it could be hypothesized that *14-3-3* might play important roles in phytohormone regulation and simultaneously mediate transduction between calcium and hormone signaling pathways [64].

The actions of plant hormones in regulating physiology and development often involve extensive cross-talk between different signaling pathways [65]. It is now clear that auxin is involved in multiple processes in developing xylem, of which many or all processes involve interactions with other hormones, including gibberellins, brassinosteroids, cytokinins, and ethylene [9,56,66]. Several reports have demonstrated that auxin and GA overlap in their regulation of multiple aspects of plant development and may mutually engage in positive cross-talk. Moreover, Björklund et al. (2007) demonstrated that GA increases auxin levels in stem by stimulating polar auxin transport [9], while Zhao et al. (2003) showed that GA shares a common transcriptome with auxin, including many transcripts related to cell growth [67]. Consistent with these results, we also found that gene expression in response to auxin signals is regulated at the transcriptional level in a manner highly similar to that of the GA response. This finding therefore provides molecular results that align with results of physiological experiments. BR and auxin control a number of similar processes, such as cell elongation and vascular development. BR and auxin positively regulate each other’s biosynthesis, resulting in a positive regulatory loop [60,68]. However, how they act together to regulate vascular development remains unclear. Moreover, BR and GA both contribute to the directional expansion of plant organs and are involved in similar developmental processes [22]. These findings add a new dimension to the concept that the endogenous IAA-GA-BR balance in plants may be determined by cross-talk between hormones. We also found that BR exerted no significant influence on most gene transcription, with only a very limited number of BR-regulated genes induced by greater than 2-fold, IAA and GA_3_ were shown to induce expression of numerous transcript levels in excess of 2-fold. Therefore, we speculate that BR-induced transcription is weak and slow in *Populus* compared with transcriptional induction by IAA and GA, as observed by others [68,69].

Notably, tissue-specific regulation of genes related to wood formation differed for different hormones, with expression of individual genes varying substantially across tissue types. For example, *KLP* expression was significantly down-regulated in leaves of plants treated with BR relative to the control, while only slight differences in expression levels were observed in stems and roots. Meanwhile, *PIN* was dramatically induced under GA_3_ treatment in stems, with no significant differences observed in roots or leaves. This would be consistent with reports that auxin transport genes in hybrid aspen polar were differentially expressed in relation to the endogenous auxin gradient as well as specific cell types during wood development [70]. We also observed tissue-specific differences whereby most gene expression in leaves was regulated by BR, with less dramatic BR-regulation in stems and roots. Shimada et al. (2003) posed that BRs were synthesized in the same tissues in which they function [71]. Consistent with this idea, Gregory M. et al. proposed that BRs do not undergo long-distance transport in pea [72]. For instance, they founded that the maintenance of steady-state BR levels in the stem does not depend on their transport from the apical bud or mature leaves. We guessed that the absence of long-distance BR transport between different plant tissues might provide significant insight into tissue-specific effects of the exogenous BR on leaves [70]. All of these results together suggest that hormonal regulation of plant growth is a a complicated regulatory process.

## 4. Materials and Methods

### 4.1. Plant. Materials and Treatments

Seedlings of *Populus simonii* × *P. nigra* were grown in pots containing a mixture of turf peat and sand (2:1 *v*/*v*) in a growth chamber under controlled conditions (temperature 22–25°C, 70–75% relative humidity, under a 14-h light/10-h dark cycle). One month later, aerial surfaces of seedlings of similar height were sprayed every 15 days with different concentrations of exogenous phytohormones. Treatments consisted of various concentrations of IAA (0.3, 3, 30, 100 mg·L^−1^), GA_3_ (10, 30, 50, 100 mg·L^−1^) and epibrassinolide (0.01, 0.1, 0.2 and 0.4 mg·L^−1^). A control treatment was applied in the same volume of ultrapure water. After four months of treatment, 18 seedlings per treatment group were collected for measurement of plant height, stem diameter and cellulose content. Stems of seedlings were harvested to permit determination of cellulose content using ANKOM A2000i analysis (A2000i, Ankom Technology Co., Fairport, NY, USA). Moreover, seedlings treated with IAA (30 mg·L^−1^), GA_3_ (50 mg·L^−1^) or BR (0.2 mg·L^−1^) were collected and subjected to anatomical measurements of biological triplicate samples.

Two-month-old untreated seedlings were sprayed with appropriate concentration levels (30, 50, 0.2 mg·L^−1^) of exogenous IAA, GA_3_ and BR, respectively. A control treatment was also applied as the same volume of ultrapure water. Following these treatments, leaves, stems and roots from seedlings were harvested after various treatment time points (0.5 h, 1.5 h, 3 h, 24 h, 2 d, 3 d, 4 d), immediately frozen in liquid nitrogen and stored at −80°C for RNA preparation. Samples for real-time PCR were prepared in triplicate.

### 4.2. Anatomical Observations

Fresh stems from comparable plant areas were fixed with FAA solution and subsequently dehydrated by passage through a series of graded ethanol solutions followed by vitrification using dimethylbenzene. Next, samples were embedded in paraffin then sectioned using a microtome. After the paraffin was removed, sections were stained with 1% safranin and 0.5% fast green and examined using a light microscope (Olympus biological microscope CX41, Tokyo, Japan). Images were captured using bright field illumination. Radial widths of xylem and phloem were measured using Image Tool software DP2-BSW (version 2.2, Olympus, Tokyo, Japan).

### 4.3. RNA Extraction and Reverse Transcription (RT)

Each plant tissue sample was separately ground into a fine powder using a mortar and pestle. Total RNA was isolated from stems using the cetyl trimethylammonium bromide (CTAB) method. For each sample, 4 μg of total RNA was digested in a 25-μl total volume with DNase I (Promega, WI, USA) to remove genomic DNA contamination. First-strand cDNA synthesis was performed using 1 μg of DNaseI-treated RNA and the Prime Script RT reagent Kit (TaKaRa, Kusatsu, Japan) according to the manufacturer’s instructions. Synthesized cDNAs were diluted 10-fold with sterile water and used as templates for quantitative reverse-transcriptase PCR (qPCR).

### 4.4. Quantitative Real-Time PCR

According to digital gene expression profiling data, 27 genes related to wood formation in *Populus simonii* × *P. nigra* were chosen for qPCR expression pattern studies. Gene expression levels in leaves, stems and roots were investigated for responses to exogenous IAA, GA_3_, and BR at 0.5 h, 1.5 h, 3 h, 24 h, 2 d, 3 d or 4 d. Gene-specific primers were designed using Primer 5.0 software and primer sequences are shown in Table 1. qPCR was carried out using an Opticon machine (Biorad, Hercules, CA, USA) with a real-time PCR MIX Kit (SYBR Green as the fluorescent dye, TOKOBO, Shanghai, China). The *TUA* (CA822230) and *UBQ* (BU879229) genes were used as reference genes to normalize total RNA amountper reaction. The 20-μL reaction mixture contained 10 μL of SYBR-Green Real-time PCR Master Mix (Toyobo), 0.5 μM of each specific primer for target genes or reference genes and 2 μL of cDNA template. Amplification was performed using the following cycling parameters: 94 °C for 30 s, followed by 45 cycles at 94 °C for 12 s, 60 °C for 30 s, 72 °C for 40 s and 1s at 81 °C followed by reading of plates. qPCR was carried out in triplicate to ensure reproducibility of results. Expression levels were calculated to determine the threshold cycle whereby the signal exceeded the background level according to 2^−ΔΔCt^. Relative expression level = transcription level under IAA or GA_3_ or BR treatment/transcription level under control conditions. The level of significance was set to |log_2_^f^°^ld change^| >1 and *p* < 0.05.

### 4.5. Correlation Network Analysis

Expression levels of 27 genes of *Populus simonii* × *P. nigra* were determined using the 2^−ΔΔC*t*^ method. A Pearson correlation method matrix was determined for all samples using 2^−ΔΔC*t*^ for all detected genes. Correlation coefficients were calculated using cor, a function of R. Gene interaction pairs with Pearson correlation coefficients greater than 0.85 were used to construct the correlation network. The network was visualized using Cytoscape version 3.6.1 (www.cytoscape.org).

### 4.6. Statistical Analysis

Analyses of variance (ANOVA) of plant height, ground diameter, cellulose content, xylem and phloem thickness were performed using SPSS software (SPSS, Chicago, IL, USA). The least significance difference (LSD) method was used to perform differential analyses between pairs of means of multiple experiments. The level of significance was set to *p < 0.05*.

## 5. Conclusions

The effects of IAA, GA_3_, and BR on plant growth, physiology, and gene expression levels in *Populus* were studied. The results indicated that IAA appeared to affect xylem development much more than phloem development, while GA mainly affected xylem fiber elongation rather than radial expansion. A total of 27 genes related to wood formation in *Populus simonii × P. nigra* were selected for expression pattern studies using qPCR. Most genes were regulated by IAA, GA_3_, and BR and showed different expression patterns under the three phytohormone treatments between leaves and stems, with the BR-induced transcription profile weak and slow compared with corresponding profiles for IAA and GA_3_. Notably, gene expression in roots exhibited similar expression patterns in response to all three phytohormone treatments. This study paves the way toward a more comprehensive understanding of molecular regulatory mechanisms involved in wood formation in response to hormone signaling in *Populus*.

## Figures and Tables

**Table 1:** Primer sequences of the 27 genes examined and reference genes in this study.

Symbol	Accession NO	Description	Primer sequences (forward)	Primer sequences (reverse)
Cell wall biosynthesis				
*CAD*	Potri.009G095800	Cinnamyl-alcohol dehydrogenase	GCATCTCGCTCCTTACACCT	TCCCACCTCAACAACCTCAC
*CCR*	Potri.009G076300	Cinnamoyl-CoA reductase	ATACCGTTCACGCCACCG	GAAAGACGCCAGCACAGC
*CesA8-B*	Potri.004G059600	Cellulose synthase *8-B*	GGCTTGCCATGAGTGTAA	ATCCTGAGAATCGTTGAG
*EXPA4*	Potri.010G202500	Alpha-expansin 4 precursor	AATCCTCCTCGTCCTCACTTC	CCTTCACGCTCACTTTCACAA
*GH*	Potri.008G120000	Glycosyl hydrolases family 31	TGGGAAGTGCCATACAATCT	TCACCAAATGACCCTGAACC
*GLA*	Potri.007G099800	Beta-galactosidase	CTGCTGCCATTCATTATCCT	TGTTTCAATCACATCCACCC
*GT*	Potri.002G200200	Glycosyl transferase family 8	AAGCCATTCACTCCTCCAA	GAGAAGCCGCATTCATCAG
*KLP*	Potri.010G153000	Kinesin-like protein	GCAGCACAATCAGAGCCTAAC	ATAACCTCCCAATGCACCACC
*PAL*	Potri.008G038200	Phenylalanine ammonialyase	AACCCAACTATTCCAAAC	CTTCAAGCATTCCAGCAT
*Pel*	Potri.015G087800	Pectate lyase	GGAGAAGAACCGTCAGAGGC	ACGGGATCATCATTACCAGAGT
*Susy*	Potri.006G136700	Sucrose synthase	AACTTCGTGCTTGAATTGGACT	AACAACTTAGCTGAAAGGTGGC
*XET*	Potri.005G201200	Xyloglucan Endotransglycosylase	GATTCTGGTTCTGGCTTCC	GTCAATCTCGTCGTGGGTC
Transcription factors				
*CCR4*	Potri.002G182500	Transcriptional effector CCR4	CTGCTAACTGCTGTGCGTAA	TTGGCATAAGGTTGAGTTTGTT
*ERF2*	Potri.001G154100	Ethylene-responsive transcription factor 2	CTTACGACCGTGCTGCCTAT	TGCCACCAACCTTCTTCCT
*Myb156*	Potri.009G134000	Myb-like DNA-binding domain	GAAGATTACCAGGGAGAACAGAT	CTGAAGTAGTAGTCGTGGTGAAAG
*PPR*	Potri.011G120900	Pentatricopeptide Repeat Protein	GGGTTGAAGAGGGCAAGAAA	AATCAGATCCAGCCGCACAG
*RAP2-3*	Potri.008G210900	Ethylene-responsive transcription factor	CGGAGATTGACAGTTGAGGAT	ACTTGCTGGACTTGGATGGTG
*SHINE2*	Potri.018G028000	Ethylene-responsive transcription factor	GACCCTTCTCCTTCACTCACT	CCTCTTCTTCCGTACCATTTT
Signal transduction				
*Aux/IAA 4*	Potri.013G041300	Auxin-responsive protein 4	GCCTGACATGAATGAAGAGCC	CTTGATGGGTGGAGCAGTTTC
*Aux/IAA 14*	Potri.008G161200	Auxin-responsive protein 14	ATGGAGCCCAGGGAATGATAG	AGGATGGCACATACTCGGAAC
*CABP*	Potri.016G024700	Calcium binding protein	ATAAGGATGGCGATGGTTGC	AGCCTCAGTCGGGTTCTGTC
*CaM*	Potri.012G041000	Calmodulin-like protein 6a	GAATGGCTTTATTTCTGCTGC	CATCCTCACAAACTCCTCGTA
*GIP*	Potri.017G083000	Gibberellin regulated protein	GCTGTCTTCCTCTTGGCTCT	GGCTTATGGTACTGGGTCTTG
*PIN*	Potri.015G038700	Auxin efflux carrier component 1	ACCATTACATTGTTCTCGCTTTC	GAGATGAGCAGTTTAGCACCC
*PP2A1*	Potri.010G054300	Serine/threonine protein phosphatase 2A	CTGGTCTTGATTCTGCTCCTC	GCAATGCTTCATACGGTGG
*PP2A2*	Potri.003G015400	Serine/threonine protein phosphatase 2A	CTTCGCCATCCCAACATAAT	CTCGTCTTCGCTGAATCGTC
*14-3-3*	Potri.T147900	14-3-3 protein	GGCTCCTACCCACCCAATA	GCAAGACTGCAAGCACGAT
Reference genes				
*TUA*	Potri.003G220300	Tubulin alpha-3/alpha-5 chain	AGGTTCTGGTTTGGGGTCTT	TTGTCCAAAAGCACAGCAAC
*UBQ*	Potri.001G418500	Polyubiquitin	GTTGATTTTTGCTGGGAAGC	GATCTTGGCCTTCACGTTGT

## Supplementary Materials

Supplementary Materials can be found at http://www.mdpi.com/1422-0067/20/3/792/s1.

## Abbreviations

ANOVA	Analysis of variance
BR	Brassinosteroid
CTAB	Cetyl trimethylammonium bromide
eBL	Epibrassinolide
GA	Gibberellin
GA_3_	Gibberellic acid
IAA	Indole-3-acetic acid
Myb	Myeloblastosis
NAC	NAM–ATAF1,2–CUC2
qPCR	quantitative real-time PCR
Susy	Sucrose synthase
